# Olfactory Loss and Brain Connectivity after COVID-19: Structural Follow-Up at One Year

**DOI:** 10.1155/2023/6496539

**Published:** 2023-04-29

**Authors:** Fabrizio Esposito, Mario Cirillo, Rosa De Micco, Giuseppina Caiazzo, Mattia Siciliano, Andrea G. Russo, Caterina Monari, Nicola Coppola, Gioacchino Tedeschi, Alessandro Tessitore

**Affiliations:** ^1^Department of Advanced Medical and Surgical Sciences, University of Campania “Luigi Vanvitelli”, Piazza L. Miraglia 2, 80138 Napoli, Italy; ^2^Department of Mental and Physical Health and Public Medicine, University of Campania “Luigi Vanvitelli”, Largo Madonna Delle Grazie 1, 80138 Napoli, Italy

## Abstract

The structural connectivity from the primary olfactory cortex to the main secondary olfactory areas was previously reported as relatively increased in the medial orbitofrontal cortex in a cohort of 27 recently SARS-CoV-2-infected (COV+) subjects, of which 23/27 had clinically confirmed olfactory loss, compared to 18 control (COV-) normosmic subjects, who were not previously infected. To complement this finding, here we report the outcome of an identical high angular resolution diffusion MRI analysis on follow-up data sets collected in 18/27 COV+ subjects (10 males, mean age ± SD: 38.7 ± 8.1 years) and 10/18 COV- subjects (5 males, mean age ± SD: 33.1 ± 3.6 years) from the previous samples who repeated both the olfactory functional assessment and the MRI examination after ~1 year. By comparing the newly derived subgroups, we observed that the increase in the structural connectivity index of the medial orbitofrontal cortex was not significant at follow-up, despite 10/18 COV+ subjects were still found hyposmic after ~1 year from SARS-CoV-2 infection. We concluded that the relative hyperconnectivity of the olfactory cortex to the medial orbitofrontal cortex could be, at least in some cases, an acute or reversible phenomenon linked to the recent SARS-CoV-2 infection with associated olfactory loss.

## 1. Introduction

In our previous 3 Tesla advanced MRI study [1], using high angular resolution diffusion imaging (HARDI) tractography, we observed that the structural connectivity index (SCI) of the primary olfactory (piriform) cortex to 16 major secondary olfactory functional areas [2] was significantly increased, across both cerebral hemispheres, in the medial orbitofrontal cortex (mOFC-SCI), in a cohort of 27 recently SARS-CoV-2-infected subjects (COV+ group: 10 males, mean age ± SD: 40.0 ± 7.6 years), the large majority of which 23/27 had clinically confirmed anosmia or hyposmia, compared to an age- and sex-matched cohort of 18 healthy normosmic subjects (COV- group: 6 males, mean age ± SD: 36.0 ± 7.1 years), none of which had been previously infected with SARS-CoV-2.

Olfactory loss had been already reported in both acute and postacute phases of the COVID-19 disease [3, 4] and even six months after resolution of COVID-19 [5]. However, at the time of our baseline MRI observations, it was not possible to state whether the observed structural differences existed already before SARS-CoV-2 infection, suggesting some form of innate vulnerability of the central olfactory pathway in COV+ subjects with insulting olfactory loss, or rather these changes had developed rapidly, within two or three weeks after SARS-CoV-2 infection, together with olfactory loss, suggesting a (mal)adaptive structural response of the neural tissue. Indeed, both SARS-CoV-2 infection and acquired olfactory loss had been previously independently associated with changed cerebral morphology within core olfactory areas [6, 7].

To possibly gain additional insight into the previous data as well as on the often-postulated link between SARS-CoV-2 infection and insulting olfactory loss, which often persisted over several months, both COV+ and COV- subjects enrolled in the baseline study (between April and December 2020) were subsequently invited to participate in the 1-year follow-up study (to be completed between April and December 2021). In the reminder, we will keep the labels COV+ and COV- to refer to the recruited subjects according to the grouping established at baseline, i.e., regardless of the clinical status resulting at follow-up, albeit further subgroups will be considered to account for the possible change in their clinical conditions.

## 2. Methods

The follow-up study had been ethically approved by the Institutional Review Board of the University of Campania “L. Vanvitelli” (protocol no. 0008735), and a written informed consent had been initially obtained from all subjects participating in the baseline study, albeit all subjects might still withdraw from the study. Indeed, only 18 COV+ subjects (10 males, mean age ± SD: 38.7 ± 8.1 years) and 10 COV- subjects (5 males, mean age ± SD: 33.1 ± 3.6 years) eventually accepted to repeat both the olfactory functional assessment and the neuropsychological and MRI examinations, according to the same clinical procedures and imaging methodology (see [1], for details) and showed up at the same MRI center after ~1 year from the first visit (range between visits: 330-574 days, median: 385 days). All subjects were not SARS-CoV-2 infected at the day of the follow-up visit, as ascertained by real-time polymerase chain reaction (PCR) for SARS-CoV-2 RNA. For COV+ subjects, the number of days since the previous COVID-19 diagnosis (prior to the baseline scan), estimated as the time between the first positive real-time PCR and the baseline scan, ranged between 29 and 93 days (52.16 ± 19.13 days). The time between the first positive real-time PCR and the second consecutive negative real-time PCR ranged between 10 and 76 days (37.38 ± 20.97 days). On the baseline scanning day, all COV+ and COV- subjects had recently (11.00 ± 5.86 days, range 1-21) undergone a real-time PCR confirming they were virus-free. Although the selection of the subjects for the follow-up study (as well as their classification as COV+ or COV-) was strictly based on (their status at) the baseline visit (April-December 2020), both COV+ and COV- subjects reported no history of COVID-19 between the two visits.

From the HARDI MRI data sets, we derived the SCI [8] from the tractography of primary olfactory (piriform) cortex to all 16 brain regions collecting potential connections from the piriform cortex. This metric provides a quantitative index of structural connectivity across the central olfactory pathways and previously yielded sufficient evidence at baseline for the relative increase in connections of the primary olfactory cortex to the medial OFC across both cerebral hemispheres. Thus, the entire procedure for estimating the mOFC-SCI was identically replicated for baseline and follow-up individual data sets, independently for each cerebral hemisphere, as described in the previous report (see [1] for all details about the structural connectivity analysis pipeline).

At the follow-up visit (on the scanning day), according to the classification proposed by Hinz et al. [9], 10/18 COV+ subjects, who were found anosmic or hyposmic at baseline (i.e., Sniffin's test score below 10), were confirmed as anosmic or hyposmic (COV+ subgroup 1, *n* = 10), whereas 6/18 COV+ subjects, who were found anosmic or hyposmic at baseline, were found normosmic (Sniffin's test score equal to 11 or 12) (COV+ subgroup 2, *n* = 6). All 10/10 COV- subjects were confirmed normosmic at follow-up. Two COV+ subjects, who had been previously classified as normosmic, were found hyposmic at follow-up. All COV+ and COV- subjects were confirmed as cognitively unimpaired at the neuropsychological testing performed at follow-up (age- and education-adjusted MoCA score higher than the Italian cut-off of 15.5 points; for more details, see [1]).

The statistical analysis of the SCI measures was performed in MATLAB R2022b (Mathworks, Inc., http://www.mathworks.com) using functions from the statistics toolbox. The correction for covariates (i.e., age and sex adjustment) was performed for the measures of all subjects (separately for baseline and follow-up data) via linear regression (function: “regress”). The test-retest reliability of the SCI measure for this longitudinal study was estimated by pooling all measures from COV- subjects (*n* = 10) and computing the intraclass correlation coefficient (ICC) between baseline and follow-up data. For the testing of differences between (sub)groups, given the small number of observations in each (sub)group, the signed rank test (function: “signrank”) and the rank sum test (function: “ranksum”) for equal medians were, respectively, applied to compare the age- and sex-adjusted mOFC-SCI between two independent (sub)groups (at baseline and follow-up) and between paired observations (baseline vs. follow-up) in each (sub)group. For all tests, the critical threshold was set to 0.05 without correction for multiple comparisons.

## 3. Results

The test-retest reliability of the age- and sex-adjusted mOFC-SCI metric in the COV- group (*n* = 10, two measures) was good (ICC = 0.63; see, e.g., [10]).

**Figure 1:**
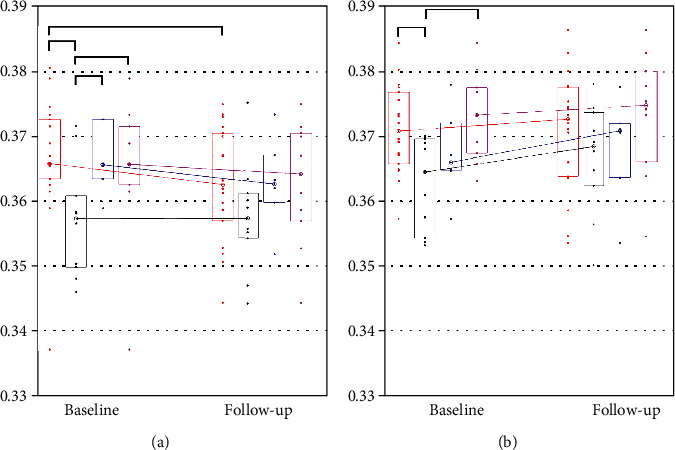
Distribution of the SCI metric obtained for COV+ groups (and subgroups) and for the COV- group, at baseline and follow-up. (a) Left medial OFC. (b) Right medial OFC. The medians and the interquartile ranges are displayed as lines and boxes for COV+ group (red, *n* = 18), COV- group (black, *n* = 10), COV+ subjects with resolved olfactory loss (blue, *n* = 6), and COV+ subject with persisting olfactory loss (magenta, *n* = 10). Horizontal black segments indicate which groups showed statistically significant differences in the distributions (*p* < 0.05).

Figure 1 summarizes the distribution (median and interquartile range) for the age- and sex-adjusted mOFC-SCI metric in COV+ groups (and subgroups) as well as in the COV- group, at baseline and follow-up. From these graphs, opposite trends were noted for the median value of the mOFC-SCI in COV+ and COV- subjects: namely, COV+ subjects had reduced (left) or unchanged (right) median values at one-year follow-up, compared to COV- subjects, and a similar trend was noted for both COV+ subgroups (persisting and resolved olfactory loss).

Using nonparametric statistics, we confirmed that, at baseline, the median of the age- and sex-adjusted mOFC-SCI was significantly and bilaterally increased for the restricted group of COV+ subjects (*n* = 18) who participated in both the baseline and the follow-up study, compared to the restricted group of COV- subjects (*n* = 10) who participated in both the baseline and the follow-up study (two-sided rank sum test: left mOFC rank sum = 317, *p* = 0.0078; right mOFC rank sum = 308, *p* = 0.026). This was not the case for the follow-up observations (two-sided rank sum test: left mOFC rank sum = 290, *p* = 0.17; right mOFC rank sum = 286, *p* = 0.24). Indeed, at least for the left hemisphere, there was a significant reduction in the same index for the COV+ group from baseline to follow-up (two-sided signed rank test: left mOFC signed rank = 134, *p* = 0.035; right mOFC signed rank = 93, *p* = 0.74) that was sufficient to restrict the variability of this metric within the corresponding range observed for COV- subjects at the same time point.

The same index was also significantly higher at baseline (two-sided rank sum test: left mOFC rank sum = 132, *p* = 0.045; right mOFC rank sum = 137, *p* = 0.017) and not any longer significantly different at follow-up (two-sided rank sum test: left mOFC rank sum = 121, *p* = 0.24; right mOFC rank sum = 126, *p* = 0.12), for the subjects belonging to COV+ subgroup 1 (i.e., subjects with persisting olfactory loss), compared to the COV- group. Similarly, albeit only for the left hemisphere, the index was significantly higher at baseline (two-sided rank sum test: left mOFC rank sum = 72, *p* = 0.022; right mOFC rank sum = 59, *p* = 0.43) and not any longer significantly different at follow-up (two-sided rank sum test: left mOFC rank sum = 63, *p* = 0.23; right mOFC rank sum = 54, *p* = 0.79), for the subjects belonging to the COV+ subgroup 2 (i.e., subjects with resolved olfactory loss), compared to the COV- group. There were not any significant differences between the two COV+ subgroups, at either baseline (two-sided rank sum test: left mOFC rank sum = 84, *p* = 0.99; right mOFC rank sum = 99, *p* = 0.15) or follow-up (two-sided rank sum test: left mOFC rank sum = 85, *p* = 0.99; right mOFC rank sum = 99, *p* = 0.15).

## 4. Discussion

The purpose of this study was to longitudinally assess the SCI metric, for the structural connectivity of medial orbitofrontal cortex relative to all major primary and secondary olfactory brain areas, in a restricted group of subjects who underwent a highly specific HARDI-MRI data analysis, both shortly and about one year, after their recovery from COVID-19.

Apart from our previously published study on the baseline data of larger groups [1], there are currently no comparable (cross-sectional or longitudinal) studies of the olfactory brain structural connectivity that were conducted with a similar approach in patients who have had COVID-19 (with or without olfactory loss) and healthy controls with normal olfaction and (reportedly) no history of COVID-19. Indeed, all previous structural MRI studies in COVID-19 patients employing diffusion MRI have mostly investigated the morphology of the white matter, and even when tractography was performed, this was mainly used to define the anatomical tracts for averaging voxel-based diffusion imaging metrics, such as diffusion tensor metrics (see, e.g., [11]), or more advanced HARDI metrics, such as kurtosis and neurite orientation dispersion and density (see, e.g., [12]). Nonetheless, the large effect size observed in the baseline analysis (albeit on larger groups), but also the good test-retest reliability of the HARDI-MRI metric, as estimated in the present group of COV- subjects across two time points, was encouraging about the possibility of running a longitudinal analysis on the same metric, albeit all presented results should be interpreted with extreme caution because of the low sample size of both the COV+ and COV- groups.

The results of the longitudinal analysis indicate that the abnormal increases in the mOFC-SCI observed in COV+ (vs. COV-) subjects were (i) only evident at baseline, i.e., in the early aftermath of SARS-CoV-2 infection, and (ii) not any longer evident at 1-year follow-up, whether these subjects were still found clinically hyposmic or had ceased to clinically manifest this symptom. As none of the subjects had a history of olfactory impairment before SARS-CoV-2 infection, we may thus speculate that the likelihood for these differences being present already before SARS-CoV-2 infection in COV+ subjects (a scenario deemed plausible in the previous discussion on baseline findings) is certainly reduced in the light of these new findings. In fact, if the baseline effect was any indication of a chronically or congenitally altered brain olfactory connectivity in COV+ subjects, i.e., independent of the insulting SARS-CoV-2 infection with associated olfactory loss, we should have seen such an effect more preserved after one year of follow-up, at least in those COV+ subjects with persistent olfactory loss. In contrast, it remains equally plausible that the infection might have actually stimulated an acute structural plasticity response of the brain olfactory connectivity, which was initially (i.e., shortly after infection) stronger and more tightly associated with olfactory symptoms, but later (i.e., after several months) progressively slower, until restoring an approximately normal connectivity pattern, definitely uncoupled from the actual olfactory performance. This scenario would be in line with a recent longitudinal study on the global structural connectivity of former hospitalized COVID-19 survivors suggesting that adverse effects on brain functioning and structure would abate over time [13]. Thus, while much larger samples would be needed for a proof of this scenario, it is now less likely that the SCI would work as a prognostic tool to identify individuals whose COV+ related olfactory loss will resolve or persist over a term as long as one year.

In conclusion, the follow-up data seem to indicate that the relative hyperconnectivity of the olfactory cortex with respect to the medial OFC detected at baseline might be, at least in some cases, an acute or reversible phenomenon linked to the previous SARS-CoV-2 infection with associated olfactory symptoms. About one year after the infection, this central olfactory brain connectivity alteration might either disappear or become less evident, with the structural connectivity index of the medial OFC falling within the normal variability of subjects who (presumably) never experienced a clinically manifest olfactory loss between the two time points (albeit they might have been infected during one of the pandemic waves occurred between the second half of 2020 and the first half of 2021). Thus, future brain connectivity studies with larger sample sizes will also require multiple control groups to eventually clarify whether the central nervous system is indeed capable of producing an acute or reversible structural connectivity response within the olfactory pathway explaining the appearance or the persistence of the olfactory loss symptoms after SARS-CoV-2 infection.

## Data Availability

The conditions of our ethics approval did not permit public archiving of study data. Anonymized data will be shared by request from any qualified investigator from the corresponding author. Access will be granted to named individuals in accordance with the ethical conditions governing the reuse of sensitive data.
